# Multivariate meta-analysis reveals global transcriptomic signatures underlying distinct human naive-like pluripotent states

**DOI:** 10.1371/journal.pone.0251461

**Published:** 2021-05-13

**Authors:** Kory R. Johnson, Barbara S. Mallon, Yang C. Fann, Kevin G. Chen

**Affiliations:** 1 Intramural IT and Bioinformatics Program, National Institute of Neurological Disorders and Stroke, National Institutes of Health, Bethesda, Maryland, United States of America; 2 NIH Stem Cell Unit, National Institute of Neurological Disorders and Stroke, National Institutes of Health, Bethesda, Maryland, United States of America; Johns Hopkins School of Medicine, UNITED STATES

## Abstract

The ground or naive pluripotent state of human pluripotent stem cells (hPSCs), which was initially established in mouse embryonic stem cells (mESCs), is an emerging and tentative concept. To verify this vital concept in hPSCs, we performed a multivariate meta-analysis of major hPSC datasets via the combined analytic powers of percentile normalization, principal component analysis (PCA), *t*-distributed stochastic neighbor embedding (*t*-SNE), and SC3 consensus clustering. This robust bioinformatics approach has significantly improved the predictive values of our meta-analysis. Accordingly, we revealed various similarities or dissimilarities between some naive-like hPSCs (NLPs) generated from different laboratories. Our analysis confirms some previous studies and provides new evidence concerning the existence of three distinct naive-like pluripotent states. Moreover, our study offers global transcriptomic markers that define diverse pluripotent states under various hPSC growth protocols.

## Introduction

The concept concerning the ground or naive states of pluripotent stem cells was initially proposed by Smith and colleagues [1]. Naive pluripotent stem cells found in preimplantation mouse embryos are distinguished from lineage-primed epiblast stem cells (EpiSCs) derived from post-implantation mouse embryos [2, 3]. Thus, mouse pluripotent stem cells have at least two distinct (primed and naive) states. The maintenance of the naive state relies on the use of leukemia inhibitory factor (LIF) with two inhibitors, GSK-3βi and ERK1/2i (abbreviated as 2i), which suppress glycogen synthase kinase-3β (GSK-3β) and extracellular signal-regulated kinases 1/2 (ERK1/2), respectively. Conceivably, pluripotent stem cells in the naive state may have several potential advantages over those with the primed state, particularly for facilitating single-cell growth, genetic manipulation, disease-modeling, drug discovery, and multi-lineage differentiation (reviewed in references [4–8]).

Here, we define mouse embryonic stem cells (mESCs) derived from the preimplantation embryos as naive pluripotent stem cells. Those cells derived from human embryos or converted from human pluripotent stem cells (hPSCs), which recapitulate all or part of mESC features, are referred to as naive-like pluripotent stem cells (NLPs). In the past seven years, several groups have reported the conversion of primed hPSCs, which depend on distinct growth signals that embrace FGF2/Activin-A/TGFβ signaling pathways, to NLPs and *de novo* derivation of NLPs from the human inner cell mass [9–18]. However, there is a lack of robust assays that precisely define a naive pluripotent state under different growth conditions *in vitro*. The existing assays used for determining pluripotent and differentiation states mainly count on various genome-wide analyses [9, 10, 12, 13, 19]. However, genome-wide transcriptomic levels across datasets generated from different laboratories using different technologies (e.g., microarray and RNA-sequencing) often have substantial differences in expression scale and spread. A direct meta-analysis of the transcriptomic levels across datasets can render confusing results and lead to incorrect interpretations and conclusions. Accordingly, previous genome-wide data analyses revealed significant differences between various NLPs derived from different laboratory protocols [12, 13], hence confounding the definition of human naive pluripotency. Thus, there is a pressing need to address the above critical issues.

In this study, we employed a meta-analysis approach that integrates genome-wide microarray and RNA sequencing (RNA-seq) data into the principal component analysis (PCA) [20], *t*-distributed stochastic neighbor embedding (*t*-SNE) [21], and SC3 consensus clustering [22]. We aim to resolve critical interlaboratory experimental inconsistencies with human naive pluripotency. Our integrated approach significantly reduced the interferences of the batch effects on interlaboratory data analysis. With this approach, we characterized transcriptomic signatures of NLPs from publicly available datasets, and systematically evaluated data from current human naive-like protocols. Our analysis revealed the existence of distinct naive-like pluripotent states in both converted and derived NLPs, which are deficient in global transcriptomic signatures in early human embryos. Our study also provides new insights into the role of 1D- and 2D-meta-analysis in gene cluster rearrangements, thereby enabling us to define accurate pluripotent states.

## Materials and methods

### Datasets for meta-analysis

We collected 12 datasets for multivariate meta-analysis [9–12, 15–17, 23–26]. These datasets are composed of 265 samples from 9 independent laboratories (S1 Table). The datasets can be identified with GSE and EMBL-EBI accession numbers in parentheses: D3 (E-MTAB-2031), D5 (GSE46872), D6 (GSE50868), D7 (GSE59435), D22B (E-MTAB-2857), D23 (E-MTAB-2856), D24 (E-MTAB-4461), D25 (GSE36552), D26 (GSE29397), D27 (SRP115256), D28 (GSE44430), and D29 (GSE141639). We curated these datasets based on their laboratories, first author(s), the size of samples (n), cell types (e.g., blastocysts and hESCs), cellular states (e.g., primed or naive-like), culture medium with growth factors, protocols, feeder/coating (e.g., MEFs or Matrigel), oxygen tension (e.g., normoxia and hypoxia), and RNA processing platforms (e.g., microarray and RNA-seq) (S1–S3 Tables). Of note, there are substantial differences in the cell culture protocols used to generate and maintain hPSCs between individual laboratories, particularly in the methods used to derive naive-like hPSC (S2 Table). Another major protocol difference is the use of oxygen. For example, the naive-like cells in D3 and D6 were cultured under normoxia, which contradicts most NLPs grown under hypoxia. Moreover, the primed hPSCs in three datasets (D3, D6, and D27) were maintained in either Matrigel (D3 and D6) or vitronectin (D27) coated plates (S2 Table). The remaining primed hPSCs and all NLPs were cultured on MEF feeders. All detailed information can be found in Supporting information.

### RNA-seq and microarray datasets for validation of normalization methods

We used two validated intra-laboratory cDNA microarray and RNA-seq datasets, which presumably had minimal laboratory protocol differences, from the laboratories of Dr. Austin Smith and Dr. Elias Zambidis. These datasets can be briefly described as follows: D22B (E-MTAB-2857, RNA-seq; primed H9 cell lines, n = 3; H9 Reset lines, n = 3), D23 (E-MTAB-2856, microarray; primed H9 cell lines, n = 3; H9 Reset lines, n = 9), D24 (E-MTAB-4461, RNA-seq; HNES1-3 Reset cell lines, n = 9), D28 (GSE44430, microarray; primed hPSCs, n = 7; NLPs, n = 7), and D29 (GSE141639, RNA-seq; primed hPSCs, n = 4; NLPs, n = 6). More detailed information is available in S1 and S2 Tables.

### Data transformations for meta-analysis

**Fig 1:**
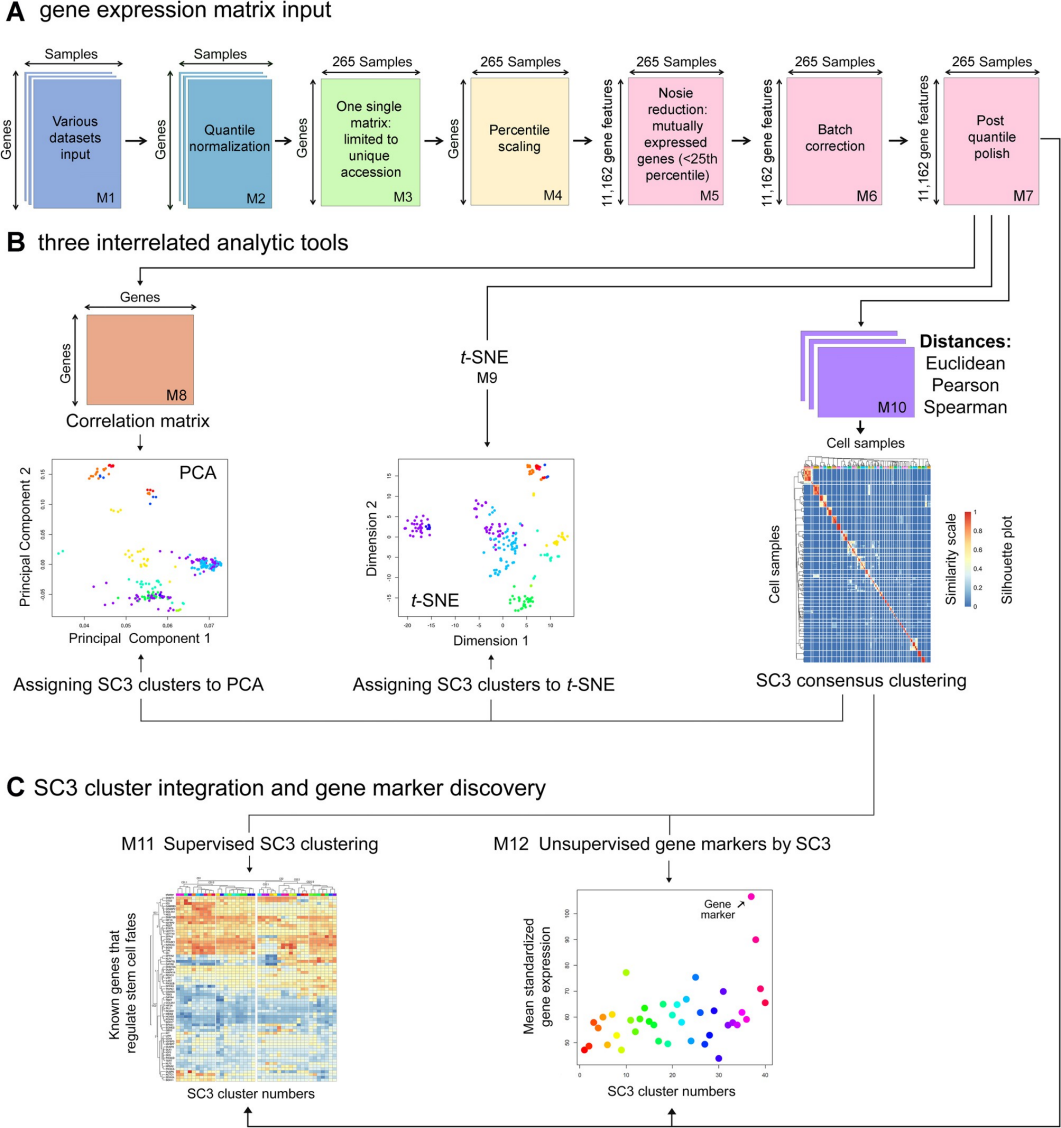
Scheme of a multivariate meta-analysis. The meta-analysis platform consists of 12 independent modules (M1-12) that constitute three major components. (A) Gene expression matrix input. The matrix comprises 12 datasets and 265 cell samples. Data are transformed by multiple consecutive steps (i.e., quantile normalization, limited to unique genes, collapsing median values, and percentile scaling followed by the exclusion of mRNA noise and batch correction). Regarding the inclusion or elimination of unique genes, we followed the following steps: (i) utilizing Ingenuity to standardize and assign the approved Human Genome Organization (HUGO) symbols to each dataset, (ii) filtering genes for each dataset, which could not be assigned with a HUGO symbol, (iii) identifying the unique union set of HUGO symbols across all datasets, (iv) collapsing redundant genes within each dataset, (v) constructing a single gene expression matrix using the unique union set of HUGO symbols (n = 11,162), (vi) flooring the gene expression values below the 25^th^ percentile, and (vii) filtering genes not having at least one sample from one dataset with a percentile value greater than 25. (B) Three interrelated analytic tools (i.e., correlation PCA, *t*-SNE, and SC3 consensus clustering) for the transformed datasets; The Silhouette plot, a quantitative measure of the diagonality of the SC3 consensus matrix, is based on *k*-means clustering. (C) SC3 cluster integration into PCA and *t*-SNE (upper panel) and gene marker discovery modules (lower panel). Known regulatory genes of the pluripotent states, integrable with SC3 consensus clusters, are used as controls to validate the above meta-analysis approach and differentiate stem cell states.

Meta-analysis was used to analyze the above 12 datasets and compare various pluripotent states (Fig 1A). To enable different datasets to be used for meta-analysis, we transformed all datasets by the following major steps: (i) quantile normalization within datasets [27], (ii) data filtering to retain unique genes only, (iii) data collapsing methods to calculate the median values of multiple gene probes, and (iv) percentile coding (from 1 to 100%) of each gene expression in each sample followed by removing mutually expressed genes below the 25^th^ percentile.

Briefly, we performed quantile normalization (Log_2_) of the transcriptome per dataset (total datasets = 12) using *ad-hoc* commands in the statistical programming language R (http://cran.r-project.org/), creating a master expression matrix of 11,162 unique genes in rows and 265 samples in columns. The expression levels recorded in this matrix by all samples were further coded by quantile bins (1–100%). Explicitly, the observed expression values across all genes for a sample were used to define the 1^st^ to 100^th^ quantile values. These quantile values were then used to code where each expression value for the sample falls. For example, if an expression value for a gene of the sample falls between the 20^th^ and 30^th^ quantile values, the gene expression then has a defined value of 20. This quantile-bin approach was applied to all genes per individual sample.

Consequently, the ranking of RNA expression within a sample for a dataset was preserved, resulting in the transformed values ranging from 1 to 100. These transformed values could then be used to compare the ranks of RNA expression across all datasets. However, we further removed those percentile values below the 25^th^ percentile to boost high confidence signals without a substantial bias. As indicated in the Tukey box plot, the differences in data distribution and location were grossly apparent. Accordingly, we used “removeBatchEffect” function from the “limma” package in R to implement batch correction [28]. The Tukey box plot was further used to verify whether the differences across the datasets were maximally reduced. Minor differences were amended by quantile normalization (S4 Table). Finally, the datasets were used for meta-analysis based on correlation PCA, *t*-SNE, and SC3 consensus clustering, aiming to reveal the significant influencing factors that control cellular and pluripotent states.

### PCA

The percentile-normalized and mRNA noise-removed datasets were used to construct a Pearson correlation-based matrix (*A*, a gene expression correlation versus gene expression correlation matrix) that accounts for 100% variations of gene expression profiles. The inverse correlation matrix (*A*^*-1*^) was employed to calculate the principal components (PCs, known as eigenvectors with the largest eigenvalues) (e.g., PC1, PC2, and PC3) by orthogonal decomposition using R programming. For example, PC1, PC2, and PC3 represent the sum of weighted correlation (*W*_*i*_) for each gene expression correlation (*G*_*i*_) in each sample (*S*_*j*_) in one column. Thus, these PC values were used to map the data points in PCA scatter plots, in which each data point contains the genome-wide gene expression correlation profile of one sample (or cell type).

### *t*-SNE visualization and cluster analysis

The *t*-SNE analysis was implemented by the R program based on a curated meta-information table. To generate *t*-SNE plots, quantile “polished” datasets were first pedestalled by 1 and log2-transformed, and then passed to the “Rtsne” function under default parameters (dims = 2, perplexity = 30, theta = 0.5, pca = T, momentum = 0.5, final momentum = 0.8, eta = 200). Individual samples can be differentiated by colored clusters in plots. For example, we can assign colors to all samples based on the names of datasets (e.g., D3, D5, and D23) and pluripotent states (e.g., naive-like and primed). Of note, the *t*-SNE plots may differ from each other using the same datasets for analysis at a different time [21].

### SC3 consensus clustering

Several clustering packages (e.g., CrossICC, RCC, and SC3) have been developed for cross-platform analysis [22, 29, 30]. Both CrossICC and RCC are similar to SC3 in terms of the consensus similarity matrix generated by applying multiple clustering algorithms (e.g., agglomerative clustering, k-means, and custom algorithms). SC3 has gained increasing popularity, owing to the method that is based on Euclidean, Pearson, and Spearman distances for the consensus clustering. Hence, this method does not have the shortcomings of using any one of the three metrics alone. Technically, SC3, a user-friendly R package (Bioconductor version 3.12, http://bioconductor.org), accepts the data input as an expression matrix with observations in columns and gene expression in rows. It can be used for both single-cell and bulk RNA-seq data analysis [22].

To increase the strength of the PCA and *t*-SNE analysis, we integrated SC3 consensus clustering into PCA and *t*-SNE plots to identify transcriptomic signatures in the subsets of cells or cell groups (Fig 1B). The SC3 package includes the function named “sc3_plot_consensus” that allows us to evaluate the sample-cluster relationship for a selected number of clusters (*k*). We explored sample versus cluster assignments over a wide range of *k* using this function. We found *k* = 40 to arguably be the best one, which provided a diagonal consensus matrix that was neither over- nor under-clustered.

To determine top gene markers in each cluster, we also applied the above “sc3” function to export both differentially expressed features across the samples (regardless of cluster assignments) and candidate gene markers per cluster. The mean cluster expression values (under all cell samples of each cluster) were used to construct a binary classifier prediction for a given gene. By default, features with an area under the receiver operating characteristic (AUROC) curve were used to quantify the accuracy of the classifier prediction of gene markers in SC3 clusters. Through the Wilcoxon signed-rank test, a *P*-value was given to each gene (S5 Table). To define a gene marker, AUROC was set to 0.8 with a 0.05 *P*-value threshold. Different AUROC values (ranging from 0.8 to 0.99) can be explored to examine the robustness of gene markers in each cluster (S5 Table).

To better view the relationship between SC3 clusters and the pluripotent states, we integrated the supervised into unsupervised analyses, introduced cluster breakpoints, and aligned the clusters with cellular or pluripotent state indicators (Fig 1C). The cluster fragments may provide a quick view of the interchangeability of cluster rearrangements. The outcomes of gene cluster rearrangements largely depend on the size(s), function, redundancy, and the numbers of SC3 gene clusters.

### Web resources used in the study

https://www.ncbi.nlm.nih.gov
http://bioconductor.org/packages/release/bioc/vignettes/SC3/inst/doc/SC3.html
https://distill.pub/2016/misread-tsne/
https://www.genecards.org
https://www.uniprot.org

## Results

### Assembly of a meta-analysis platform for differentiating pluripotent stem cell states

We have established a multivariate meta-analysis by integrating a rigorously normalized data matrix into PCA, *t-*SNE, and SC3 consensus clustering. This meta-analysis platform consists of 12 independent modules (M1-12) that build up to three main components (Fig 1). These components include one gene expression matrix, three interrelated analytic tools (i.e., PCA, *t*-SNE, and SC3 consensus clustering), and two gene marker discovery modules (i.e., SC3 cluster integration into PCA and *t*-SNE) (Fig 1). One of the biggest challenges of this meta-analysis was integrating RNA-seq datasets (n = 6) with microarray datasets (n = 6) for comparative analysis. Besides a routine quantile normalization, we utilized an additional percentile normalization followed by a percentile noise exclusion step, batch correction, and post-quantile polish (S4 Table). This integrated approach allows the comparison between the datasets with mutually expressed genes greater than the 25^th^ percentile.

### Integration of RNA-seq data with microarrays for meta-analysis reveals three distinct naive-like pluripotent states

To further validate the percentile normalization method, we used corroborated intra-laboratory datasets (D22B, D23, and D24) from Dr. Austin Smith’s laboratory, which were related to RNA-seq and microarrays (S1 and S2 Tables). These closely related datasets serve as reasonable controls to validate our normalization methods. We initially calculated the correlation coefficient (*R*) between D22B and D23 or D24 cell samples pre- and post-percentile normalization to include all possible sample pairs for comparison (S1 Fig). We found that the correlation (*R*_*pre*_ = 0.55) between D22B (H9 cell line, RNA-seq) and D23 (H9 cell line, microarray) gene expression was significantly increased in post-percentile normalized datasets (*R*_*post*_ = 0.80, *R*_post_—*R*_pre_ = 0.25; Welch modified *t-*test, *P*-value = 7.2E-37). Similar results were also found when comparing H9 Reset cell lines (S1 Fig).

Evidently, there was a high pre-percentile correlation (*R*_pre_ = 0.87) between D22B (H9 Reset cell lines, RNA-seq) and D24 (HNES1-3, RNA-seq datasets). However, this correlation was actually decreased (*R*_post_ = 0.79, *R*_post_—*R*_pre_ = -0.082; Welch modified *t*-test, *P*-value = 4.0E-16) post-percentile normalization between these RNA-seq datasets (S1 Fig). Thus, these results suggest that some RNA-seq processing methods may overdrive the correlation of these datasets. However, this seemingly high correlation can also be attenuated by our percentile normalization.

**Fig 2:**
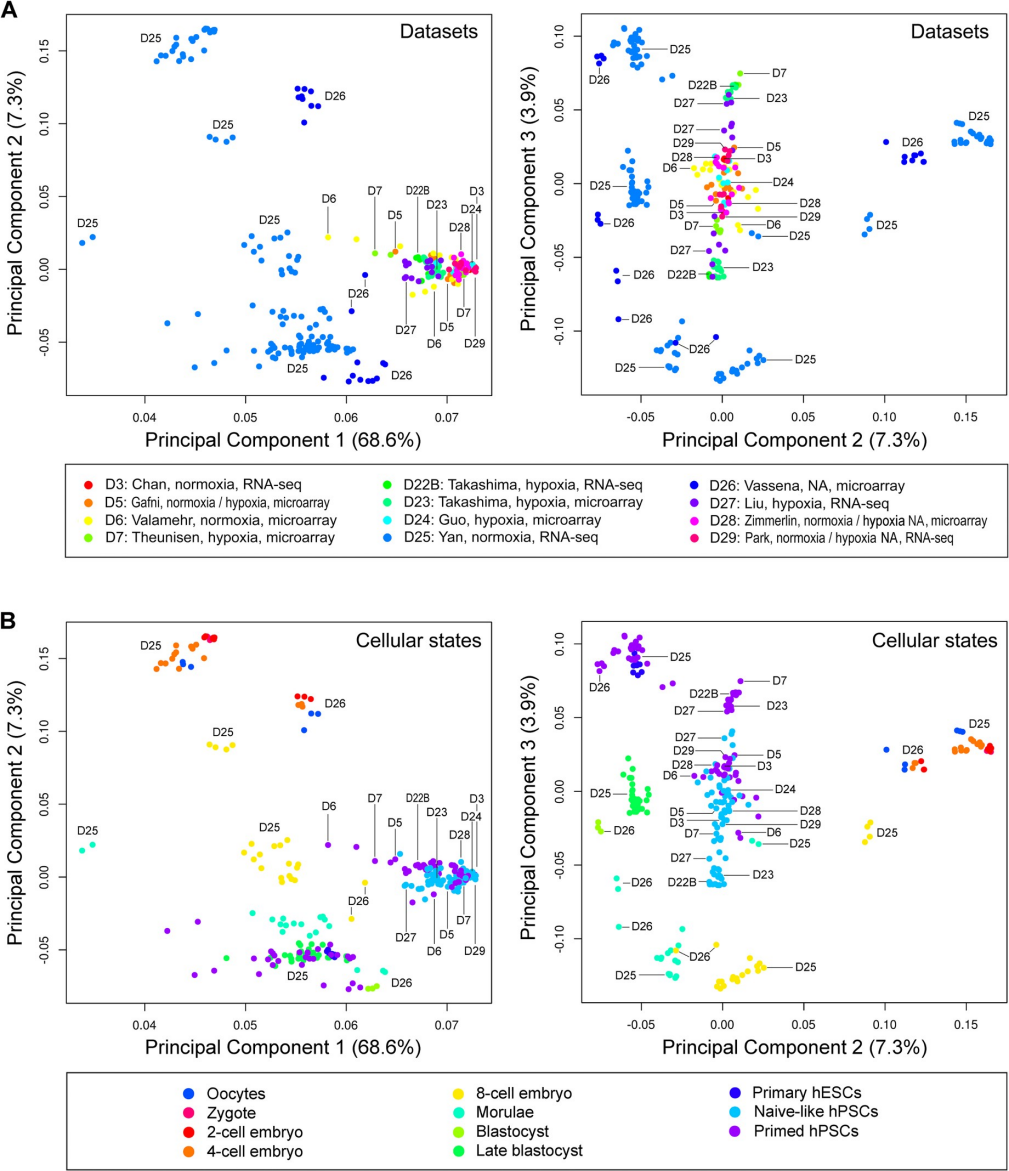
Principal component analysis (PCA) for defining pluripotent and cellular states. (A) PCA plots labeled with dataset identification numbers. The 12 datasets used in this study were named based on the first authors of the published reports. The datasets are composed of 265 samples from 9 independent laboratories, which can be identified with GSE and EMBL-EBI, and Sequence Read Archive (SRA) accession (see Materials and methods). Oxygen concentrations used for cell culture and RNA processing platforms are indicated in the lower panel. Of note, normoxic culture conditions in D25 were provided by Dr. Fuchou Tang. (B) PCA plots showing cellular states (colored dots) are labeled with dataset identification numbers. Abbreviations: NA, information not available.

**Fig 3:**
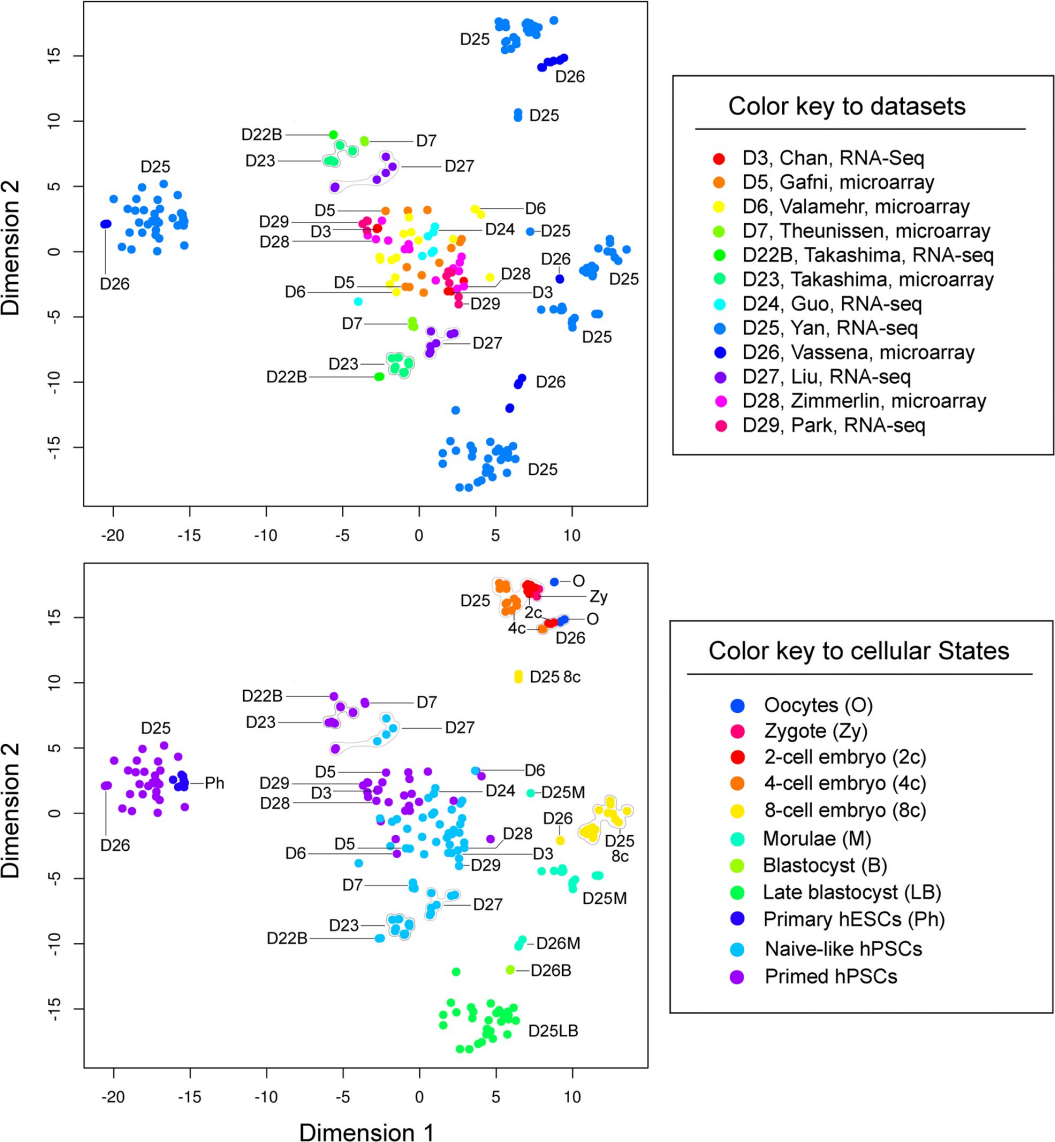
*t*-distributed stochastic neighbor embedding (*t-*SNE) plots. (A) *t*-SNE plots labeled with dataset identification numbers. (B) *t*-SNE plots showing cellular states (colored dots) are labeled with dataset identification numbers.

To further verify the comparability between RNA-seq and microarray gene expression, we performed both PCA and *t-*SNE. We focused on three paired comparisons (i.e., D22B versus D23, D28 versus D29, and D25 versus D26). Both D22B (Takashima microarray) and D23 (Takashima RNA-seq) datasets were from the Smith laboratory. The primed hPSCs and NLPs in these two datasets were separated and exhibited distinct cellular states, in which either primed hPSCs or NLPs were tightly grouped in three major principal components (i.e., PC1-3) and two *t*-SNE dimensions regardless of the significant differences between the RNA-seq and microarray platforms (Figs 2A and 2B and 3A and 3B). Similar results were also reproduced using D28 (Zimmerlin microarray) and D29 (Park RNA-seq) datasets from the Zambidis laboratory (Figs 2A and 2B and 3A and 3B). Moreover, even D25 (Yan RNA-seq) and D26 (Vassena microarray) datasets, generated from two different laboratories, are closely oriented in both PCA and *t*-SNE plots (Figs 2A and 2B and 3A and 3B). For example, human oocytes and early embryos (2-cell to 4-cell stages), which require no (for oocytes) or minimal cell culture (for early embryos) and negligible laboratory protocol differences (S2 Table), are closely associated or grouped in both PCA and *t*-SNE plots (Figs 2 and 3). PCA revealed that these oocytes and human early embryos were grouped or overlapped in the three major principal components (i.e., PC1-3), particularly in PC2 and PC3 (Fig 2B) and *t*-SNE (Fig 3) plots that depict little variability in gene expression. Taken together, the post-percentile normalization method followed by the exclusion of mRNA noise and batch correction significantly improves the comparability between RNA-seq and microarray datasets, which may be suitable for the analysis of datasets from different platforms in one meta-analysis.

Thus, with our rigorous approach, we were able to reveal a progressive cellular state transition from morulae toward blastocysts, naive-like, and primed pluripotent states (Figs 2 and 3). Moreover, our meta-analysis defines primed hPSCs and NLPs with multiple distinct groups, likely with distinct pluripotent states (Figs 2A and 2B and 3A and 3B). There were three separate groups for NLPs based on their similarities to early human embryos (e.g., blastocysts or late blastocysts) and primed hPSCs in PCA and *t*-SNE plots. Group I NLPs, generated by t2iLGö (D22B, D23, and D27) and 5iLA (D7 and D27) from three independent laboratories, clustered adjacent blastocysts and late blastocysts. Group II NLPs, with an intermediate state between Group I and primed hPSCs, comprised six datasets (D3, D5, D6, D24, D28, and D29) generated by five different protocols (S2 Table). Within this group, NLPs from D6 showed a scattered distribution, implying heterogeneous pluripotent states. Group III NLPs, derived from two different protocols in one dataset (D27), displayed a remarkable similarity to primed hPSCs than Group I and II NLPs (Figs 2A and 2B and 3A and 3B).

**Fig 4:**
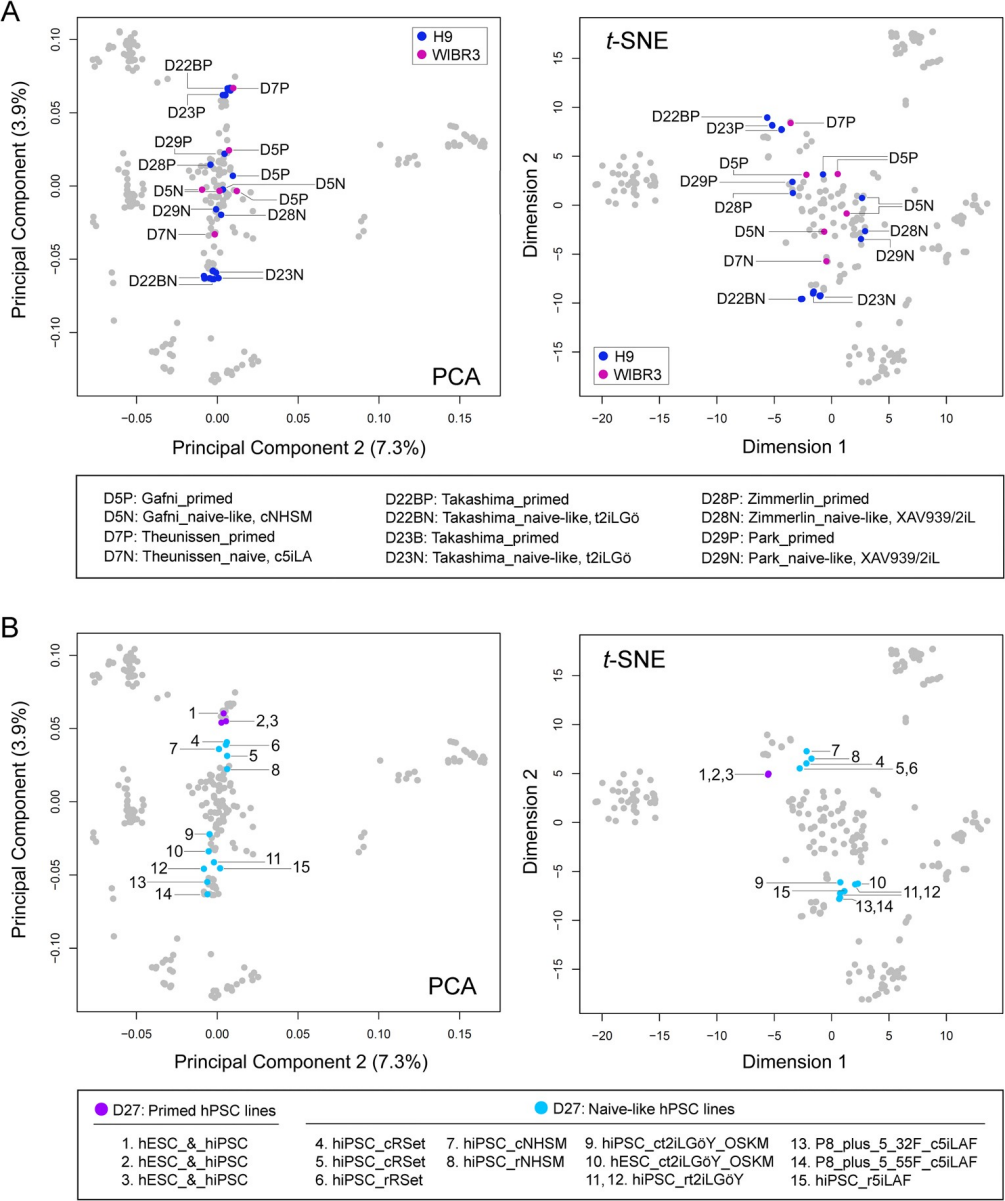
PCA and *t*-SNE plots. (A) Assessing interlaboratory cellular heterogeneity (in H9 and WIBR3 hESCs). The cell samples are labeled with dataset identification numbers (e.g. D5 and D7) and the primed (P) or naive (N) pluripotent state. For example, D5P denotes cell samples from D5 with the primed pluripotent state. (B) Examining intra-laboratory pluripotent state variations attributable to protocol differences. Both primed and naive-like cell samples (D27), derived from multiple different protocols in the Polo laboratory, were used to validate our analytic approach. Abbreviations for hPSC culture protocols: 2iL, the MEK and GSK3 inhibitors with leukemia inhibitory factor (LIF); 5iLA: The MEK, GSK3, ROCK, BRAF, and SRC pathway inhibitors in the presence of LIF and Activin; 5iLAF, 5iLA in the presence of FGF2; c, conversion of a pluripotent state with medium; NHSM: Naive human stem cell medium; OKSM, the combination of the human pluripotent factors OCT4, SOX2, KLF4 and c-MYC; r, reprogramming; RSet: RSet medium from StemCell Technologies Inc.; t2iLGӧ: Titrated MEK and GSK3 inhibitors with LIF plus the PKC inhibitor Gӧ6983; t2iLGӧY: t2iLGӧ in the presence of the ROCK inhibitor Y-27632; XAV939/2iL: 2iL in the presence of the tankyrase inhibitor XAV939.

Noticeably, distinct NLPs found in D5 and D27 were contributed by the same protocol (based on NHSM) implemented in different laboratories. This discrepancy could be explained in part by using cell lines with diverse backgrounds for analysis. For example, H9 and WIBR3 hESCs have different genetic backgrounds. In both PCA and *t*-SNE studies, primed H9 hESCs in D22B and D23 were distinguished from H9 hESCs in D5, D28, and D29. Similarly, WIBR3 cells in D5 are different from those in D7 (Fig 4A). Nevertheless, distinct NLPs can be generated with diverse protocols in one single accredited laboratory to minimize the influence of the genetic background of cell lines, RNA processing platforms, and laboratory-specific discrepancies on data variability [16, 18]. Notably, such distinct naive-like pluripotent states derived from multiple different protocols (e.g., RSet, NHSM, t2iLGöY, and 5iLAF) could be faithfully identified using our current methods (Fig 4B) [16]. Thus, these results reinforce the reliability of our analytic tools for accurately defining cellular states based on global transcriptomic signatures.

### SC3 clustering unveils multiple clusters that define various cellular and pluripotent states

**Fig 5:**
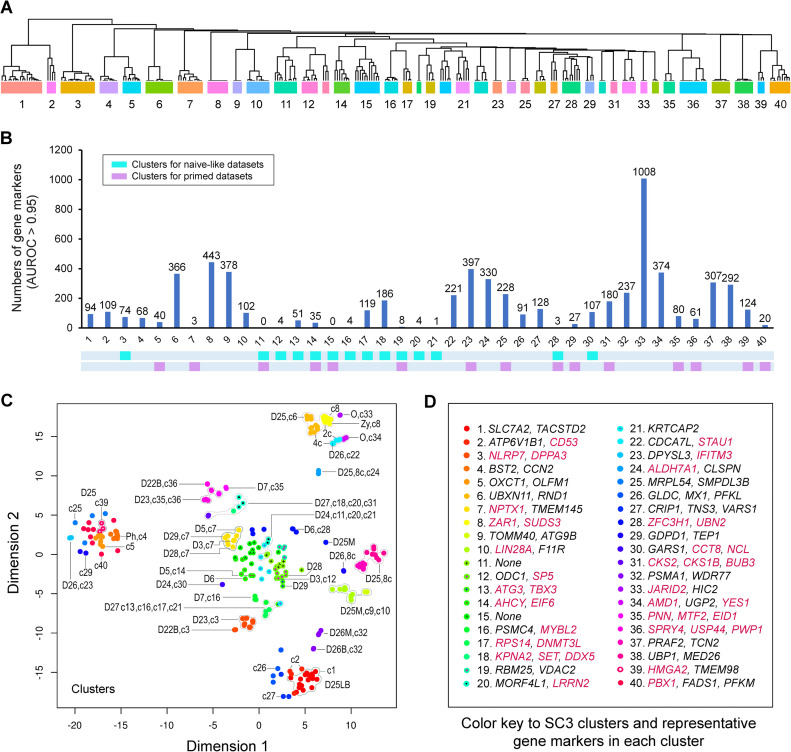
Transcriptomic clustering, gene marker identifications, and *t*-SNE integration. (A) 40 SC3 clusters were based on Euclidean, Pearson, and Spearman using SC3 consensus clustering. Shown here is the dendrogram delineating the similarities among 40 SC3 gene expression clusters. The whole heatmap is available in S2 Fig. (B) Histogram that summarizes the numbers of gene markers in 40 SC3 clusters, which are defined by *P*-values (< 0.05) and the area under receiver operating characteristic (AUROC > 0.95). Cyan- and purple-colored blocks are used to denote the clusters defining naive-like and primed states, respectively. (C) Co-assignment of 40 SC3 clusters (c1-40) with dataset identification numbers (e.g., D3) into *t*-SNE plots. (D) Color key to SC3 clusters with representative gene markers found in the top-15 gene marker list for each individual cluster. Gene markers with known functional roles in the regulation of pluripotency and embryonic development are labeled in red-colored gene symbols.

To provide a better resolution of cellular or pluripotent states, we constructed a heatmap composed of 40 SC3 consensus clusters across all datasets after post-percentile normalization (followed by mRNA noise exclusion and batch correction) [22]. The dendrogram delineates the similarities or dissimilarities among these gene clusters, which were defined by a *P*-value (*P* < 0.05) and AUROC (> 0.95) (Figs 5A and 5B and S2 and S5 Table). The numbers of gene markers in all clusters range from 0 to 1008, with a total of 6,304 gene markers among the 40 clusters (Fig 5B and S5 Table).

All SC3 clusters were assigned into the *t*-SNE plot for discriminating transcriptomic features among all datasets (Fig 5C). Noticeably, multiple clusters (e.g., C4, C5, C25, C29, C39, and C40) may define different primed pluripotent states in primary hESCs and early passages of hESCs (D25) (Fig 5C). Similarly, the four distinct SC3 clusters (i.e., C1, C2, C26, and C27) depict three major groups of cells in the late blastocyst stage (Fig 5C), which are consistent with their cellular dynamics in this embryonic stage [25]. Therefore, SC3 clustering combined with *t*-SNE likely offers a precise way to assess cellular states for large interlaboratory datasets, which have significant systemic variations.

To further evaluate the relative specificity of top gene markers among the 40 clusters, we calculated each gene marker’s mean standardized expression in all samples under each cluster. It appears that the top gene marker expression displays a significant fluctuation/scatter among the 40 clusters (S3 Fig). It is yet unknown how the vast majority of these top gene markers in each cluster regulate the fates of pluripotent stem cells both *in vivo* and *in vitro*. Hence, we unbiasedly surveyed the top 15 gene markers in each cluster of interest (Fig 5D and S5 Table), focusing on those having curated known functions in reliable informatics (www.ncbi.nlm.nih.gov, www.genecards.org, www.uniprot.org). Collectively, there are 12 gene clusters (i.e., c3, c12-14, c16-21, c28, and c30), characteristic of *in vitro* cell types with naive-like states (Fig 5D and S5 Table).

With regard to SC3 clusters defining the datasets with naive-like states, C3, characteristic of naive-like cells derived from the t2iLGö protocol in D22B and D23, possesses numerous gene markers of interest among the top 15 gene markers (Fig 5D and S5 Table). These gene markers are directly involved in the regulation of naive pluripotency (e.g., *DPPA3*) [31, 32], modulating TGFβ-mediated transcription via association with SMAD proteins (e.g., *SNW1*), controlling the mitochondrial homeostasis and apoptosis of mammalian embryos (e.g., *DAP3*) [33], and governing mouse early embryonic development at the blastocyst stage (e.g., *TGS1/PIMT*) [34].

Noticeably, c12 and c13 define naive-specific hPSCs in D27, D28, and D29. At least ten genes (e.g., *SP5*, *ATG3*, *TBX3*, *AHCY*, and *EIF6*) are associated with the regulation of pluripotency and the development of preimplantation embryos (Fig 5D and S5 Table). Besides some previously known naive gene markers such as *TBX3*, the *AHCY* gene is of interest. It encodes adenosylhomocysteine hydrolase, a new protein marker in the mouse preimplantation blastocyst. Adenosylhomocysteine hydrolase has been implicated in the regulation of embryonic stem cell proliferation [35].

Moreover, c16-18 characterize the gene clusters specific to the naive-like state in D7 and D27, in which the NLPs were generated by using the 5iLA naive protocol (Fig 5C and 5D and S5 Table). The top gene markers in these clusters embrace *PSMC4*, *MYBL2*, *RPS14*, *DNMT3L*, *KPNA2*, *SET*, *and DDX5*, all of which are implicated in the regulation of the pluripotent states. *PSMC4*, in c16, is believed to be responsible for modulating proteasome activity in hESCs. *MYBL2* (MYB proto-oncogene like 2), also in c16, is required for the initiation of somatic reprogramming and essential for ESC chromosomal stability [36, 37]. Likewise, *RPS14* (ribosomal protein S14), in c17, is critical for ESC differentiation. Significantly, *DNMT3L* (a DNA methyltransferase 3-like enzyme) represents a typical naive pluripotent gene marker. Finally, with the top 15 gene markers of c18: KPNA2 (karyopherin-alpha 2) epitomizes an Oct4-associated protein [38]; *SET* serves a nuclear proto-oncogene essential for embryonic development [39]; and DDX5 functions as an RNA helicase involved in inhibiting reprogramming to pluripotency [40].

Jointly, c19-21 are associated with the primed state in D6 and the naive-like states in miscellaneous datasets (D5, D6, D24, D27, and D28). However, only one gene marker *(LRRN2*, leucine-rich repeat neuronal 2*)* was implicated in the regulation of pluripotency and differentiation [41]. The majority of these gene markers (n = 12), including *RBM25*, *MORF4L1*, *HNRNPR*, and *KRTCAP2*, in the regulation of pluripotency and embryonic development, are poorly characterized.

Interestingly, c30 represents one independent cluster that describes the naive state in D24, generated by using the 3iLGӧ protocol, similar to t2iLGӧ, in the Smith laboratory. At least two gene markers, *CCT8* and *NCL*, were identified within the top 15 candidate gene markers (Fig 5D). CCT8 regulates the proteostasis and immortality of hPSCs [42], whereas *NCL* encodes nucleolin that regulates early development and ESC identity [43]. Taken together, SC3 clustering readily identifies dataset-specific gene markers and potential regulators or mediators, which control ESC pluripotency and embryonic development.

### Tabular tools for predicting cellular states

**Fig 6:**
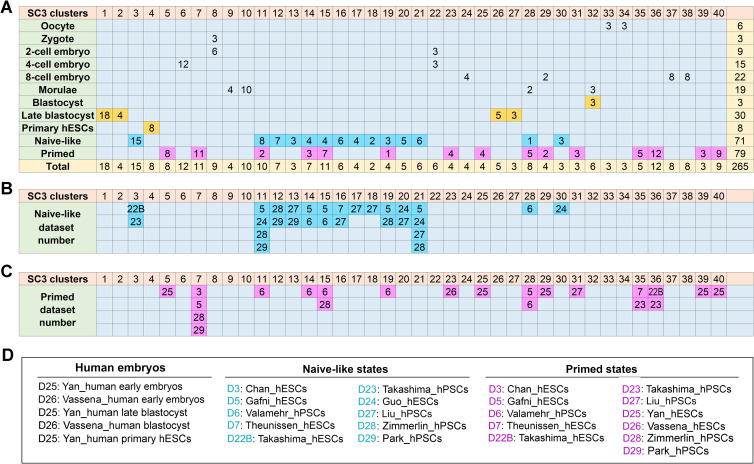
Cluster-state-dataset associations. (A-C) Tabular tools for predicting the relationships between different cellular (e.g., oocyte and zygote) and pluripotent (e.g., naïve-like and primed) states, all of which are associated with the 40 SC3 clusters in 12 datasets. Here is a hint to interpret the expression of different gene clusters in this tabular presentation: If we compare the naive-like state in one dataset (e.g. D23 in B) with the primed state in the same dataset (in C), the two states are separated by 32 clusters, thus suggesting that naive-like and primed hPSCs in D23 have a substantial difference in their actual cellular states. (D) Detailed descriptions of the pluripotent states that are associated with individual datasets. Of note, one dataset may contain two or more different cellular states, which can be distinguished by different font colors. For example, 22B is labeled with two different colors (cyan and purple), in which 22B in cyan denotes the naive-like state, whereas 22B in purple designates the primed state. Abbreviations: hESCs, human embryonic stem cells; hPSCs, human pluripotent stem cells that include hESCs and induced pluripotent stem cells (hiPSCs); Naive-like, naive-like pluripotent state; Primed, primed pluripotent state.

To reveal the complicated relationship between different cell types (e.g., oocyte and zygote), early human embryos (2-cell to late blastocysts), and pluripotent (e.g., naive-like and primed) states, we linked cellular state data to both SC3 clusters and the cellular identities. As shown in Fig 6, all cellular and pluripotent states can be assigned into 40 SC3 clusters. Our comparative analysis confirms the significant heterogeneity among naive-like and primed states (Fig 6). For example, the naive-like datasets clustered significantly differently. There were three naive-like states (Fig 6A and 6B). One was associated with late blastocysts and primary hESCs in D25. Another one was linked to the intermediate clusters c11-21 in miscellaneous datasets (Fig 6B). The third state seems to be associated with c28 and c30 (Fig 6B).

Clearly, the NLPs and primed hPSCs in one single dataset (e.g., D23) can be separated by multiple clusters (Fig 6B and 6C). Moreover, nine naive-like clusters (c3, c12, c13, c16-18, c20, c21, and c30) present discretely, whereas five naive-like clusters overlap with primed groups (Fig 5B and 5C). Among the D5, D6, D24, D28, and D29 datasets, some cell samples with the naive-like states share SC3 clusters with primed states, suggesting that these naive-like hPSC samples have a closer relationship with primed hPSCs. Briefly, our tabular analysis indicates that the naive-like state in D22B and D23 is significantly different from other naive-like states described in this study, including that of D24 generated from the same laboratory (Fig 6B). Thus, these results demonstrate that our analytic approach can differentiate between naive-like and primed states.

### Supervised cluster analysis defining new gene signatures for characterizing pluripotent and cellular states

**Fig 7:**
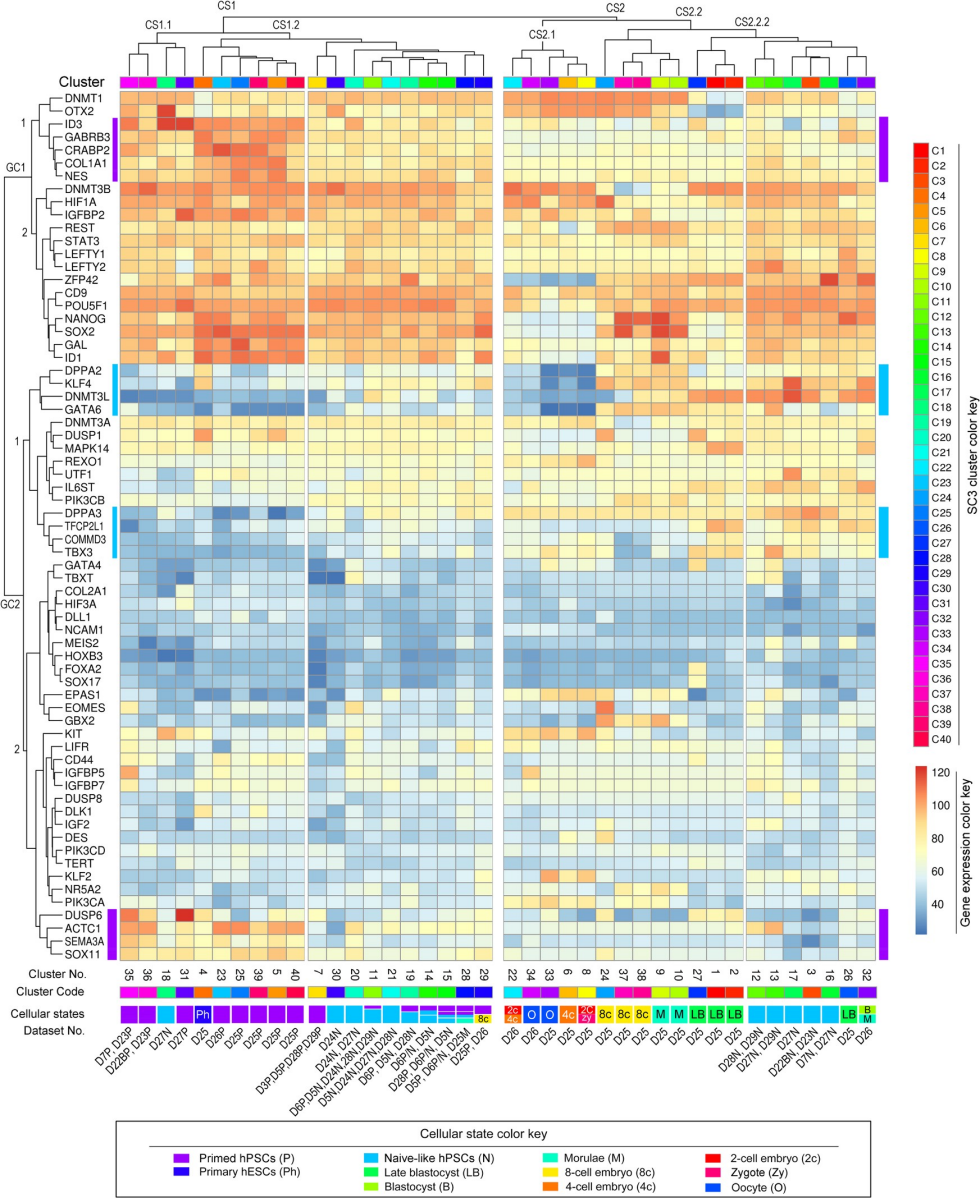
Supervised gene cluster analysis of cellular states. The heatmap is generated with the normalized mean expression for supervised gene markers from all samples under each defined SC3 cluster. Shown here is the heatmap of normalized mean gene expression across all SC3 clusters (n = 40) for supervised (known) gene markers (n = 67), in which dominant signaling pathways (n = 22), differentiation marker (n = 17), developmental regulators (n = 18), and pluripotency transcriptional factors (n = 10) were used for the process of SC3 clustering algorithms. Of note, unsupervised SC3 employs 11,122 unique gene markers for clustering analysis (Fig 5 and S5 Table). Both cellular states and datasets associated with SC3 clusters are labeled on the bottom of the heatmap. Each cluster contains one or more cell types or states as indicated by color coding detailed in the lower panel. Additional abbreviations: GC, gene cluster; CS, cellular state cluster, No., number.

In addition to the above-unsupervised meta-analysis, we also present here a supervised heatmap that delineates normalized mean expression of 67 known gene markers (www.genecards.org) (Fig 7). These notable genes encode dominant signaling molecules (n = 22), differentiation markers (n = 17), developmental regulators (n = 18), and key pluripotency transcriptional factors (n = 10). The heatmap demonstrated new features concerning cellular and pluripotent similarities associated with the 40 SC3 clusters described in Fig 5.

We analyzed the influence of known gene markers on SC3 cluster rearrangements. As indicated by annotated major cluster breakpoints, the mean standardized expression of these markers results in two major gene clusters (GC1 and GC2) that classify the 40 clusters into two global cellular state clusters (CS1 and CS2) (Fig 7). These major clusters were further divided into several subclusters for facilitating data analysis. Clearly, all naive-like and primed clusters appear to be driven by the overexpression of one gene cluster, including *DNMT3B*, *HIF1A*, *IGFBP2*, *REST*, *STAT3*, *LEFTY1/2*, *ZFP42*, *CD9*, *POU5F1*, *NANOG*, *SOX2*, and *GAL*, in the GC1.2 block (Fig 7).

Concerning the influence of developmental regulators on SC3 clustering, three major DNA methyltransferase genes (i.e., *DNMT1*, *DNMT3A*, and *DNMT3B*) showed ubiquitously high levels of mRNA expression in all clusters (Fig 7), thus diminishing their predictive values for the pluripotent states. Furthermore, the *Xist* and *ESRRB* transcripts, two of the developmental hallmarks of naive pluripotency, were not included in this analysis due to the mRNA noise exclusion step. Nonetheless, the CS2 cluster and its associated subclusters define human oocytes and early embryos from Vassena and Yan datasets [25, 26]. Upregulation of the four naive gene markers (i.e., *DPPA2*, *KLF4*, *DNMT3L*, and *GATA6* under the GC2.1 subcluster) is a strong indicator that separates the mixed cellular states (that contain zygotes, oocytes, 2-cell and 4-cell embryos) from 8c-embryos, morulae, and late blastocysts.

Remarkably, the predictive values for the naive-like states underlie the expression pattern of an 8-gene cluster within the GC2.1 block, which includes *DPPA2*, *KLF4*, *DNMT3L*, *GATA6*, *DPPA3*, *TFCP2L1*, *COMMD3*, and *TBX3* (Fig 7). This 8-gene cluster has been well-established for defining the naïve-like pluripotent states in hPSCs. Not surprisingly, the naive-like datasets (D7, D22B, D23, D27, D28, and D29) generated by the t2iLGö, 5iLA, and XAV939/2iL protocols had increased expression of the above 8-naïve gene cluster and concomitantly decreased expression of the primed gene markers *DUSP6*, *ACTC1*, *SEMA3A*, and *SOX11* (Fig 7). Especially, the above six datasets showed the closest relationship with late blastocysts, blastocysts, and morulae, consistent with PCA and *t*-SNE analyses. Thus, our supervised studies not only confirm the value of some previously identified gene markers (such as *KLF4*, *DNMT3L*, *DPPA3*, and *TFCP2L1*) but also validate our analytic approach to discern pluripotent states.

## Discussion

**Fig 8:**
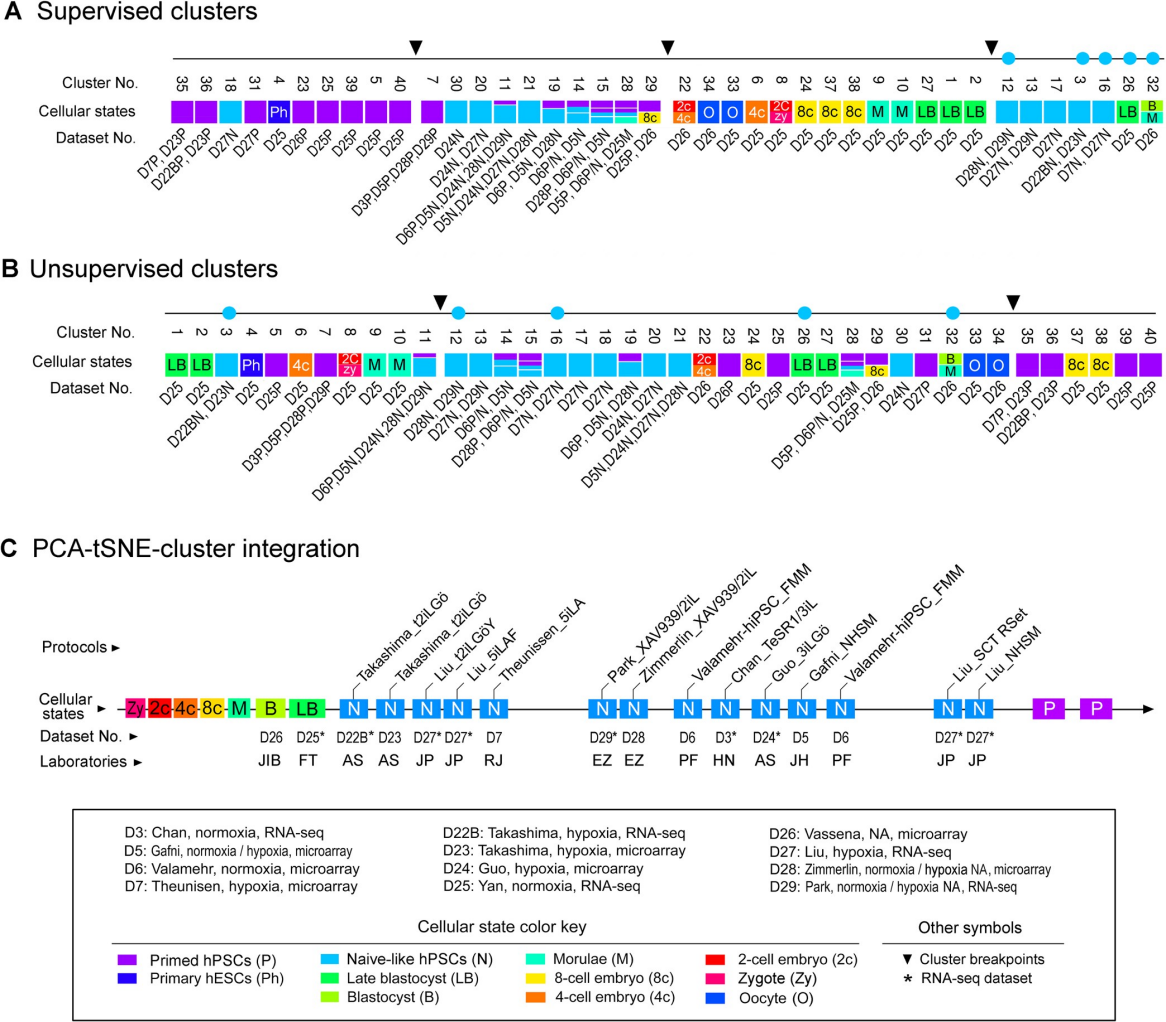
Integrated analysis of cellular states. (A and B) One-dimensional (1D) mapping of pluripotent and cellular identities by integrating supervised with unsupervised SC3 cluster analysis. Of note, supervised SC3 analysis employs 67 known gene markers, whereas unsupervised SC3 utilizes 11,122 unique gene markers for clustering analysis (Fig 5 and S5 Table). Each cluster may contain one or more cell types or states as indicated by color coding detailed in the lower panel. The clusters and cellular states are linked to the datasets that contain early human embryos, primed and naive-like hPSCs. Black arrowheads indicate cluster breakpoints that enable cluster rearrangements for better visualization of the inter-cluster relationship. The five dots in cyan are used to trace the relationship between the clusters under the different clustering conditions. (C) PCA-*t-*SNE-SC3 clustering integration for defining naive-like pluripotent states. The diagram delineates the similarities between the naive-like states in hPSCs generated from 9 independent laboratories. Human blastocysts (B) and late blastocysts (LB) are used as markers to depict the above relationship. Abbreviations for naive-like hPSC culture protocols: 2iL, MEK and GSK3 inhibitors with leukemia inhibitory factor (LIF); 5iLA: MEK, GSK3, ROCK, BRAF, and SRC pathway inhibitors in the presence of LIF and Activin; 5iLAF, 5iLA in the presence of FGF2, Chan_3iL: MEK, GSK3, and BMP pathway inhibitors in the presence of LIF; FMM: Fate maintenance medium; NA, information not available; NHSM: Naive human stem cell medium; SCT RSet: RSet medium from StemCell Technologies Inc.; t2iLGӧ: Titrated MEK and GSK3 inhibitors with LIF plus the PKC inhibitor Gӧ6983; t2iLGӧY: t2iLGӧ in the presence of the ROCK inhibitor Y-27632; XAV939/2iL: 2iL in the presence of the tankyrase inhibitor XAV939. Additional abbreviations for laboratories: AS, Austin Smith; EZ, Elias Zambidis; FT, Fuchou Tang; HN, Huck-Hui Ng; JH, Jacob Hanna; JIB, Juan Carlos Izpisua Belmonte; JP, Jose Polo; PF, Peter Flynn; RJ, Rudolf Jaenisch.

To accurately define a pluripotent state seems to be hindered by a lack of reliable analytic tools for comparative meta-analysis. Based on the integrated meta-analysis described in this study, we have provided an unbiased assessment of cellular and pluripotent states. Accordingly, we have integrated PCA and *t*-SNE with SC3 clusters that define various cellular states, including enriched panels of regulatory, metabolic, and effector gene markers (Figs 1–8 and S5 Table). Here, we will discuss the critical interference factors of multivariate meta-analysis, the rationale and reliability of our analytic approach, and the integration of supervised clustering into unsupervised analyses.

The purpose of a meta-analysis is to mitigate the interference of laboratory-specific batch effects while preserving genuine biological differences across all datasets. Batch effects, unwanted variations attributable to technical sources in high-throughput biology, substantially confound meta-analysis across different datasets. Hence, besides the interlaboratory protocol differences (S2 Table) that may derail a successful meta-analysis, other influencing batch factors (e.g., cDNA microarray and RNA-seq) should also be taken into consideration in this study. For example, when dealing with cDNA microarray and RNA-seq data, we frequently encounter: (i) sensitivity related to Poly(A) in channel versus ribosomal RNA depletion; (ii) sequencing depth (e.g., coverage, numbers of reads, and the length of reads used for mapping); (iii) paired versus single ends (in which paired ends are more accurate); (iv) stranded versus non-stranded; (v) outliers, and importantly, (vi) low levels of mRNA noise. Accordingly, some distinct SC3 clustering differences (e.g., c9, c10, and c28 versus c32 between the human morula datasets D25 RNA-seq and D26 microarray) seem to be consequential to one of the above-discussed issues (Figs 3B and 5C).

The rationale of using meta-analysis relies on validated normalization strategies that integrate interlaboratory datasets generated from different platforms. To enable impartial comparison of interlaboratory datasets, we demonstrate a new data transformation approach. That involves quantile normalization of mRNA expression across samples within each dataset, percentile coding, mRNA noise exclusion, and subsequent batch correction followed by a quantile polish step.

Our quantile normalization approach is similar to the feature-specific quantile normalization (FSQN) for cross-platform classification [44]. FSQN was used to remove RNA-seq platform bias using DNA microarray data as the target distribution. Each corresponding feature (gene) is quantile normalized by log2 transformation of RPKM counts from RNA-seq data. Moreover, we use percentile coding to preserve the relative expression abundance (ranking) within the sample. It is these rankings that can then be directly and fairly compared across datasets. Our analytic approach is also analogous to Gene Fuzzy Scoring (GFS), an emerging powerful data normalization method against batch effects [45]. By GFS, gene features are ranked and assigned with new values (ranging from 0 to 1) based on high, moderate, and low confidence gene features. The low-confidence features are considered noise and penalized by flooring the expression values to zero, similar to the analytic methods described in this study. Thus, our methods enable us to eliminate large amounts of unwanted variations and boost high confidence signals. However, excluding genes with low mRNA expression may have certain caveats, particularly to discriminating some differentiated samples (e.g., vascular progenitors, S6 Table). For example, among the all datasets, only D28 and D29 that contain CD34^+^ and vascular progenitor samples, respectively, which express a significant level of vascular genes (e.g., *CDH5*, *CD34*, *PECAM1*, *VWF*, and *PTPRC*). None of other datasets are expected to express these vascular genes at higher levels as indicated (S6 Table). These differentiation genes would be excluded for the analysis based on our current workflow. Consequently, it would be difficult to differentiate these progenitors (in D28 and D29) from other hPSC datasets. In this study, we eliminated all CD34^+^ and vascular progenitor samples from D28 and D29 so that the final datasets are more homogeneous without differentiated outliers. Thus, cautions should be taken when interpreting the results using our current workflow, which may emphasize the similarities between datasets and concomitantly display poor discrimination of cell samples with more variant gene expression.

Notably, several popular methods [e.g., ComBat and SVA (surrogate variable analysis or SVA)] have been used to correct the batch effects due to their high performance across different platforms [46–48]. However, these batch correction methods may not be suitable for our datasets by design due to numerous limitations [48–53]. In the case of this study, our collected datasets represent unique sample types, passages, protocols and are therefore mutually exclusive. Neither are batch sizes equal, nor are sample classes evenly distributed across datasets (batch groups). Under this unbalanced situation, applying certain batch correction methods may increase false-positive discoveries [52]. Specifically, SVA may reduce intra-class variability at the cost of losing sample-specific features (i.e., subpopulation effects) [48]. Subpopulations, typically under-represented in datasets, are challenging to identify, which may be biologically valuable for *in vitro* cell culture. Subpopulation effects can be removed due to that they are similar to batch effects [51]. In this study, we used a linear batch removal method, Limma [28], which seems to be compatible with our transformed datasets, significantly reducing interlaboratory data variability. The differences between the naive-like and primed states were preserved (Figs 2–8). Hence, the relationship between various cellular states is definable (Fig 8).

The reliability of this study underlies its capacities to significantly increase the comparability between RNA-seq and microarray datasets without a substantial bias (Figs 2–6 and S1), thereby generating compelling gene markers that are highly relevant to pluripotent stem cell biology. Indeed, with our analytic approach, we confirmed the similarity among NLPs (in D7, D22B, D23, and D27) generated by the t2iLGö and 5iLA protocols in three independent laboratories (Figs 2–7) [12, 16, 23]. We also identified many potentially new pluripotent markers or regulators in this analysis (Fig 5D and S5 Table). Some of these newly identified gene markers might permit us to understand the regulation of complicated human naive pluripotency. For example, a cluster of pluripotency gene markers have been identified in this study, including *AHCY*, *AMD1*, *CCT8*, *CD53*, *DDX5*, *DNMT3L*, *DPPA3*, *HMGA2*, *LRRN2*, *MYBL2*, *NLRP7*, *NPTX1*, *PBX1*, *PWP1*, *STAU1*, *SUDS3*, *TBX3*, and *ZAR1* (Fig 5D). Several gene markers (e.g., *CD53*, *DNMT3L*, *DPPA3*, and *TBX3*) identified in this analysis have shed light on the molecular basis of naive pluripotency in previous studies [11, 31, 32, 54, 55]. Other newly identified gene markers encode proteins that are also crucial for the regulation of pluripotency. For example, *PWP1*, identified from c36, encodes periodic tryptophan protein 1 homolog, a chromatin-associated factor regulating transcription. PWP1 was shown to modulate the differentiation potential of mESCs by regulating Stat3 signaling [56], which triggers one of the core regulatory circuitries of naive pluripotency [57]. This study suggests a potential role of PWP1 in the regulation of human naive pluripotency. Thus, our meta-analysis with SC3 clustering identifies biologically meaningful regulators and mediators that underlie embryonic stem cell pluripotency.

Importantly, our meta-analysis suggests that hPSCs grown under current protocols have at least three distinct naive-like pluripotent states (designated as NLP1-3). NLP1 is implicated in t2iLGö and 5iLA NLPs (in D7, D22B, D23, and D27), which show a closer resemblance to human blastocysts (Fig 8C). Moreover, NLP2 represents an intermediate naive-like state, which embraces numerous NLPs from several laboratories (Fig 8C). For example, NLPs generated by the 2iL in the presence of the tankyrase inhibitor XAV939 (D28 and D29), which are significantly different from t2iLGö and 5iLA NLPs, have been shown to promote a stable naive-like state, increase genomic stability, and improve multi-lineage functionality that is essential for regenerative medicine [6, 24]. Lastly, NLP3, close to the primed state, is highlighted by the NLPs, derived from both RSet medium (StemCell Technologies Inc.) and naive human stem cell medium (NHSM) (Fig 8C). RSet NLPs have the closest similarity to NHSM NLPs (Figs 4B and 8C). Expectedly, both RSet medium and NHSM share the same institutional origin regardless of some unknown components of RSet due to the proprietary nature of the medium. Collectively, our analysis not only confirms some results from the previous reports [12, 16] but also provides new insights into diverse naive-like states (Fig 8).

Thus, SC3 clusters can be integrated into *t*-SNE to achieve a multifaceted view of diverse pluripotent states. Integration of supervised into unsupervised analyses would have *pros* and *cons* in this meta-analysis. The supervised approach may increase the sensitivity to reveal some notable features of hPSCs or NLPs (Figs 7 and 8). However, it does not reflect cellular states at a genome-wide scale. Thus, a statement made from either a supervised (usually with a small subset of gene markers) or unsupervised analysis should be weighed differently. However, we may integrate supervised into unsupervised studies in a one-dimensional format (Fig 8A–8C). This dimensional reduction provides a quick, informative, and unbiased view of cellular and pluripotent states under various interlaboratory growth conditions. Under these circumstances, integrating SC3 clusters into PCA may provide an unbiased view of the cellular state relationship between different laboratories (Fig 8C). Our meta-analysis suggests the possibility that the previously reported similarities or differences in some naive-like cellular models are likely attributed to distinct cellular states.

Regarding PCA, the separation of interlaboratory data in the plots seems to be insufficient when including multi-laboratory datasets for the analysis (Fig 2A). This problem may be overcome by comparing PCA plots with *t*-SNE and SC3 clustering (Figs 3 and 5C). Concerning *t-*SNE visualization, the high-dimensional data reduction technique, initially developed by van der Maaten and Hinton in 2008 [21], has gained popularity for data analysis and machine learning in recent years. The most significant advantage of *t*-SNE lies in its ability to visualize data similarity in a fascinating 2D plot for high-dimensional datasets (up to thousands of dimensions). However, we should be aware that this technique is a random and non-linear method. Its dimensions, physical distances of data points, size of clusters should also be interpreted with cautions (https://distill.pub/2016/misread-tsne/). To enhance the strength of *t*-SNE in a meta-analysis, we emphasize the combined use of *t-*SNE with both PCA and SC3 clustering, as demonstrated in this study.

In summary, our meta-analysis reveals distinct naive-like pluripotent states under current naive growth protocols. There is considerable heterogeneity among various cellular and pluripotent states in a large cohort of datasets generated from different laboratories. Interlaboratory data variability is still the predominant factor that significantly limits the predictive values of meta-analysis for defining cellular and pluripotent states. The combined use of percentile normalization with PCA, *t*-SNE, and SC3 clustering, representing a new strategy to compare multiple interlaboratory datasets, has significantly improved this study’s predictive values. However, other data normalization or transformation algorithms aiming at the batch correction of interlaboratory data variations should also be considered in the future. It would be crucial for reducing interlaboratory data variability.

## Supporting information

S1 Fig Correlation between RNA-seq and microarray gene expression datasets after percentile normalization. (TIF)

S2 Fig SC3 consensus clustering and Silhouette plot. A quantitative measure of the diagonality of the SC3 consensus matrix in a Silhouette plot for 265 samples in 12 datasets, which is based on *k*-means clustering (*k* = 40).
(TIF)

S3 Fig Representative top gene markers in 40 SC3 clusters. Top gene marker presentation in the 40 unsupervised SC3 clusters. Each colored dot represents the mean standardized gene expression of all samples per cluster. Only the top 1 gene marker is labeled in the plot.
(TIF)

S1 Table Curated data information used for meta-analysis. (XLSX)

S2 Table Datasets and pluripotent stem cell culture protocols. (XLSX)

S3 Table RNA processing and platform information in datasets used for meta-analysis. (PDF)

S4 Table Gene features after batch correction and quantile polish. (XLSX)

S5 Table SC3 clusters and gene marker testing results. The numbers of gene markers in SC3 clusters (n = 40) were determined by defining the area under receiver operating characteristic (AUROC) value (> 0.80) and adjusted *P*-value (< 0.05). The numbers of gene markers can be explored based on different AUROC values (0.8 to 1), in which gene markers (defined by AUROC > 0.95, highlighted in red color) are presented in Fig 5. Abbreviations: padj, a multiple comparison adjusted *P*-value calculated for each gene marker using the Wilcoxon signed rank test; de_padj, a multiple comparison adjusted *P*-value for differentially expressed (de) genes using the non-parametric Kruskal-Wallis test, representing the likelihood of stochastically expressing a gene in one cluster over other cluster(s).
(XLSX)

S6 Table Unbalanced gene expression in datasets. (PDF)
