# Supplementary figures and images for: Multivariate meta-analysis reveals global transcriptomic signatures underlying distinct human naive-like pluripotent states
Source: PLoS One. 2021 May 13;16(5):e0251461. doi: 10.1371/journal.pone.0251461 (PMC8118304; doi:10.1371/journal.pone.0251461)

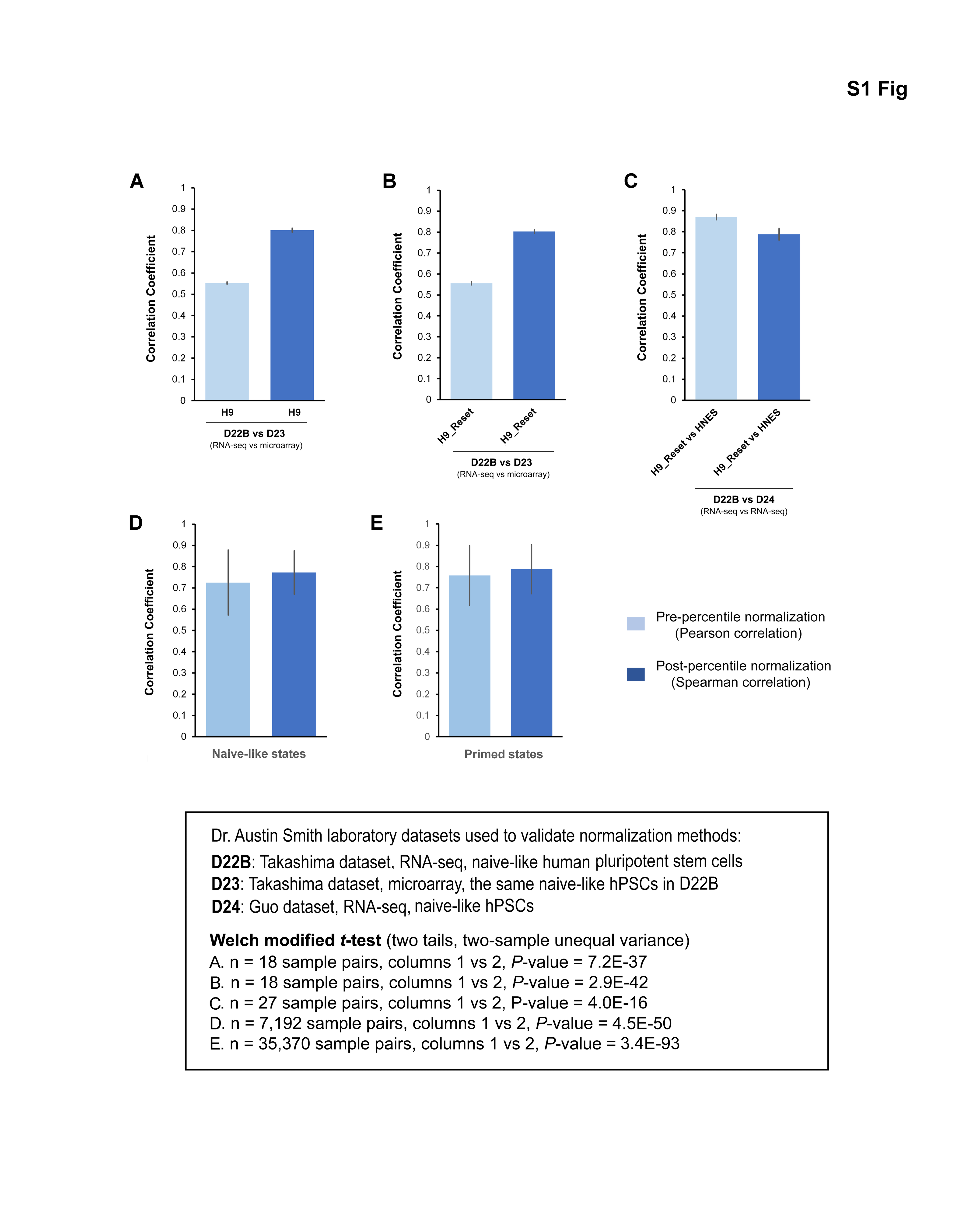

Supplement: S1 Fig — (TIF) [file pone.0251461.s001.tif]

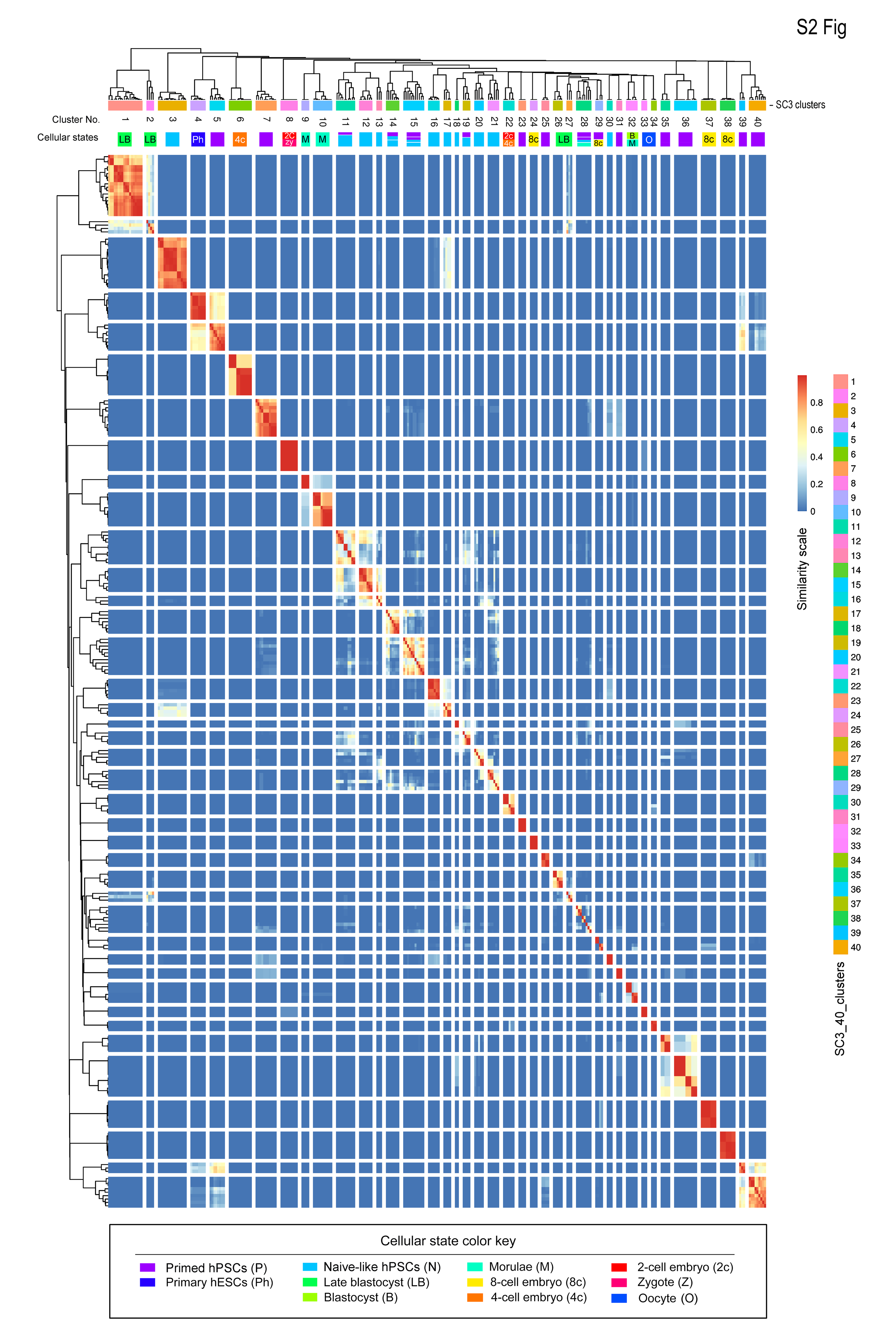

Supplement: S2 Fig — A quantitative measure of the diagonality of the SC3 consensus matrix in a Silhouette plot for 265 samples in 12 datasets, which is based on k-means clustering (k = 40). (TIF) [file pone.0251461.s002.tif]

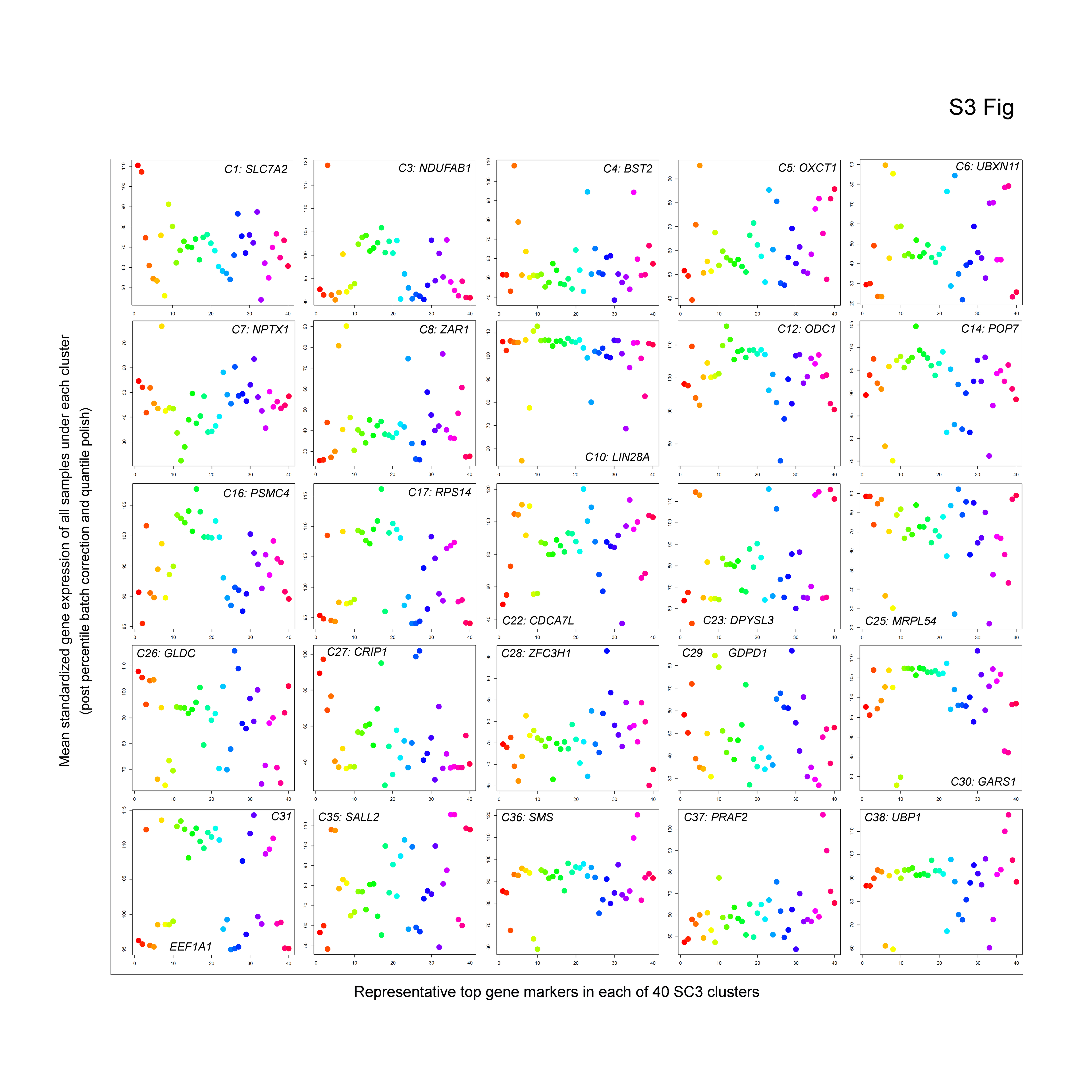

Supplement: S3 Fig — Top gene marker presentation in the 40 unsupervised SC3 clusters. Each colored dot represents the mean standardized gene expression of all samples per cluster. Only the top 1 gene marker is labeled in the plot. (TIF) [file pone.0251461.s003.tif]
