# Supplementary material for: Multivariate meta-analysis reveals global transcriptomic signatures underlying distinct human naive-like pluripotent states
Source: PLoS One. 2021 May 13;16(5):e0251461. doi: 10.1371/journal.pone.0251461 (PMC8118304; doi:10.1371/journal.pone.0251461)
Supplement: S3 Table — (PDF) [file pone.0251461.s006.pdf]

S3 Table. RNA processing information for data analysis

Dataset	Accession numbers	Expression/ Platforms	Sample RNA/ cDNA preparations	Small RNAs in samples*	Potential influence on RNA detection	Refs
D3	E-MTAB-2031	RNA-Seq<br>HiSeq 2000, Illumina	• Poly(A) mRNA	• No<br>• Less likely	May allow to measure small RNA precursors	1
D5	GSE46872	Microarray<br>Affymetrix Human Gene 1.0 ST	• Total RNAs<br>• DNA-free,<br>• No rRNA depletion	• Yes	Allow to measure small RNAs	2
D6	GSE50868	Microarray<br>Human Genome U133-plus-2.0 chips	• Total RNAs<br>• No rRNA depletion<br>• Oligo-dT-based cDNA and aRNA	• Yes	Allow to measure small RNAs	3
D7	GSE59435	Microarray<br>Affymetrix 901837	• Total RNAs<br>• DNA-free,<br>• No rRNA depletion<br>• Oligo-dT-based cDNA and aRNA	• Yes	Allow to measure small RNAs	4
D22B	E-MTAB-2857	RNA-Seq<br>Illumina HiSeq 2000	• Total RNAs<br>• DNA-free<br>• rRNA depletion<br>• Oligo-dT- and hexamer -based cDNA library	• Yes	• Sequencing read in 100-bp paired-end format.<br>• Allow to measure small RNAs and their precursors	5
D23	E-MTAB-2856	Microarray<br>Affymetrix Human Gene Array 1.0 ST arrays	• Total RNAs<br>• DNA-free<br>• Hexamer-based cRNA probe	• Yes	Allow to measure small RNAs	6
D24	E-MTAB-4461	RNA-Seq<br>Illumina HiSeq 2500	• Total RNAs<br>• DNA-free<br>• rRNA depletion<br>• Oligo-dT- and hexamer -based cDNA library	•Yes	• Sequencing read in 125-bp paired-end format<br>• Allow to measure small RNAs and their precursors	7
D25	GSE36552	RNA-Seq<br>Illumina HiSeq 2000	• mRNAs<br>• rRNA depletion (<5%)<br>• cDNAs by poly(T) primer with anchor sequence (UP1)	• No<br>• Less likely	• May allow to measure small RNA precursors	8
D26	GSE29397	Microarray:<br>Affymetrix Human Gene ST 1.0 arrays	• Total RNA<br>• DNA-free<br>• primers composed of a semi-degenerate 3' end and a universal 5' end<br>• WTA2 cDNA library	• Yes	• Product size ranges from 100–1000 bases, typically smaller for degraded RNAs<br>• Allow to measure small RNA precursors and small RNAs	9
D27	SRP115256	RNA-seq<br>Illumina HiSeq 1500 or HiSeq 3000	• RNA prepared by RNeasy micro kit	• NA	• Minimum read length of 36 bp	10
D28	GSE44430	Microarray:<br>Illumina HumanHT-12 V4.0 expression beadchip	• Total RNAs<br>• DNA-free<br>• Total RNA amplified by oligo(dT) primer	• NA	NA	11
D29	GSE141639	RNA-Seq<br>Illumina HiSeq 4000	• PolyA strand specific mRNA libraries	• NA	Using 47-bp paired-end dual indexed reads	12

Footnotes

*Both microRNA (miRNA) and small nucleolar RNA (snoRNA) precursors (i.e., pri-miRNA and pre-snoRNA), which are transcribed by RNA polymerase II and possess poly(A) tails (Lee *et al.* EMBO J 2004, 23: 4051–60; Cai *et al*. RNA 2004, 10: 1957–66; Kufel and Grzechnik P. Trends Genet 2019, 35: 104-117), can be (designated as "Yes") or cannot be (as "No" or "Less likely") preserved (or generated) using these RNA processing and reverse-transcribing methods.

Abbreviations: aRNA, antisense RNA; cRNA, antisense RNA; NA, information not available; poly(A), poly(A) mRNAs; Refs, references; rRNA, ribosomal RNA; WTA2, Whole Transcriptome Amplification Kit 2.

References: [1] Chan *et al.* Cell Stem Cell 2013, 13: 663-675; [2] Gafni *et al.* Nature 2013, 504: 282-6; [3] Valamehr *et al.* Stem Cell Reports 2014, 2: 366-81; [4] Theunissen *et al.* Cell Stem Cell 2014, 15: 471-487; [5, 6]; Takashima *et al.* Cell 2014, 158: 1254–1269; [7] Guo *et al.* Stem Cell Reports 2016, 6: 437-446; [8] Yan *et al.* Nat Struct Mol Biol 2013, 20:1131-9; [9] Vassena *et al.* Development 2011, 138: 3699-709. [10] Liu et al. Nat Meth 2017, 14:1055–1062 [11] Zimmerlin *et al.* Development 2016, 143: 4368-4380; [12] Park *et al*. Nat Commun 2020, 11: 1195.
