# Supplementary material for: Multivariate meta-analysis reveals global transcriptomic signatures underlying distinct human naive-like pluripotent states
Source: PLoS One. 2021 May 13;16(5):e0251461. doi: 10.1371/journal.pone.0251461 (PMC8118304; doi:10.1371/journal.pone.0251461)
Supplement: S6 Table — (PDF) [file pone.0251461.s009.pdf]

S6 Table. Unbalanced expression of differentiation genes (e.g., vascular genes) between pluripotent stem cells and vascular progenitor samples.

	D3	D5	D6	D7	D22B	B23	D24	D25	D26	D27	D28	D29
CDH5	True	True	False	True	True	True	True	True	True	True	True	True
CD34	True	True	True	True	False	True	True	True	False	False	True	True
PECAM1	True	False	True	True	False	False	False	True	False	False	True	True
VWF	True	True	True	True	True	True	True	True	False	True	True	True
PTPRC	False	False	False	False	False	False	False	True	False	False	True	True

Footnotes

Only D28 and D29 that contain $CD34^+$ and vascular progenitor samples, respectively, which express a significant level of vascular genes (e.g., *CDH5*, *CD34*, *PECAM1*, *VWF*, and *PTPRC*). None of other datasets are expected to express these vascular genes at higher levels as indicated in the table (S6 Table). These differentiation genes would be excluded for the analysis based on our current workflow. Consequently, it would be difficult to discriminate these progenitors (in D28 and D29) from other hPSC datasets. In this study, we eliminated all $CD34^+$ and vascular progenitor samples from D28 and D29 so that the final datasets are more homogeneous without differentiated outliers.

Abbreviations: True: gene expression that meets our criteria of data processing; False: low quality of mRNA data or mRNA expression noises, which are subject to exclusion at the step that floors the gene expression values below the 25th percentile.
